# Establishment of the retroperitoneal lymph node metastasis model of endometrial VX2 carcinoma in rabbits and observation of its metastatic features

**DOI:** 10.1186/s12957-015-0528-3

**Published:** 2015-03-14

**Authors:** Li-Qun Xu, Yong-Wen Huang, Rong-Zhen Luo, Yan-Na Zhang

**Affiliations:** Department of Gynecology, Sun Yat-sen University Cancer Center, State Key Laboratory of Oncology in South China, Collaborative Innovation Center for Cancer Medicine, 651 Dongfeng East Road, Guangzhou, 510060 People’s Republic of China; Department of Gynecology, Guangdong Women and Children Hospital, 13 Park Road, Guangzhou, Guangdong 510010 People’s Republic of China; Department of Pathology, Sun Yat-sen University Cancer Center, State Key Laboratory of Oncology in South China; Collaborative Innovation Center for Cancer Medicine, 651 Dongfeng East Road, Guangzhou, 510060 China

**Keywords:** Endometrial carcinoma, Disease models, Animal, Lymphatic metastasis, Rabbits

## Abstract

**Background:**

The objective of this study is to establish the retroperitoneal lymph node metastasis model of endometrial VX2 carcinoma in rabbits and observe of its metastatic features.

**Methods:**

The VX2 cells were transplanted into the uterine muscularis mucosae of 48 rabbits by injecting carcinoma mass suspension. According to time, the rabbits were killed after the transplantation of VX2 cells, and they were divided into six groups, 15-, 18-, 21-, 24-, 27-, and 30-day group, and six rabbits in each group. Control groups consisted of those receiving no treatment or an injection of saline. The specimens of transplanted endometrial carcinoma and retroperitoneal lymph node in the rabbits were examined histopathologically after they were killed.

**Results:**

All rabbits developed VX2 endometrial carcinoma which was confirmed with pathological examination. Significantly increased tumor volume was observed at day 24, 27, and 30 post-injection of VX2 cells (*P* < 0.05). The retroperitoneal lymph nodes were not enlarged completely in each rabbit in the 15-day group, partly enlarged in the 18- and 21-day group, and all enlarged in the 24-, 27-, and 30-day group. The histopathological examination revealed no complete retroperitoneal lymph node metastasis in the 15- and 18-day group, partial metastasis in the 21-day group, and complete metastasis in the 24-, 27-, and 30-day group.

**Conclusions:**

The model was established successfully by injecting carcinoma mass suspension, and various retroperitoneal lymph node metastasis model of endometrial VX2 carcinoma can be established rapidly in a month after the transplantation.

## Background

Endometrial cancer (EC) is one of the most common malignancies of the female reproductive system [1]. Whereas patients with early-stage EC have a good prognosis, those with advanced EC usually develop retroperitoneal lymph node (RLN) metastasis with a 5-year survival rate ranging from 30% to 40% [2-4]. Because RLN is next to large blood vessels in the abdominal cavity, once RLN metastasizes and involves the blood vessels, it increases the difficulty of surgical resection. Radiotherapy treatment is poor if lymph node size is too large, while intravenous chemotherapy drug concentration reaching low also affects prognosis. How to treat RLN metastasis is a hot spot in current research. Due to the limitation of tissue and blood drawn, it is not suitable for research in the human body; at present, a great deal of studies about RLN metastasis is conducted on animals. The VX2 cell line is composed of squamous cell carcinoma cells derived from Shope virus-induced papilloma in rabbits. Their high survival rates make them a suitable candidate for *in vivo* inoculation. This experiment is mainly to establish the retroperitoneal lymph node metastasis model of endometrial VX2 carcinoma in rabbits and observe its metastatic features.

## Methods

### Animals

A total of 49 female New Zealand white rabbits weighing 2 to 2.5 kg were purchased from the Huadong Xinhua Experimental Animal Center in Guangzhou (License No: 0098816). One rabbit was used as the source of VX2 tumor cells while the remaining animals were used to establish the *in vivo* model. The animals were individually housed, allowed free access to standard laboratory food and water, and subjected to a daily 12-h light-and-dark cycle. The animal protocol used in this study was approved by the Center’s Animal Welfare Committee.

### VX2 cells

Blocks of VX2 cells, which were kindly provided by the Cell Bank of the Sun Yat-sen University, were cut into 0.5- to 1-mm pieces and mixed in 5 mL RPMI 1640 (Life Technologies, Carlsbad, CA, USA). One milliliter of the VX2 solution (20%, approximately 1 × 10^10^ cells/mL) and 0.2 mL of VX2 were injected into the quadriceps femoris of one rabbit. After 21 days, a solid mass was removed from the injection site, washed in normal saline, and placed in RPMI 1640. Areas with active growth were selected, and the tissue was cut into pieces of 0.5 to 1 mm in diameter. After vortexing, the solution (1 × 10^10^ cells/ml) was transferred to a syringe with a lumbar puncture needle.

### Establishment of an animal model of EC with RLN metastasis

Animals in the experimental group were anesthetized with 3% pentobarbital sodium at 1 mL/kg via the ear vein and then placed in a supine position. After sterilization, a mid-line incision was made on the lower abdomen. After the uterus was exposed, 0.5 mL of the VX2 solution was injected into the muscularis mucosae of the myometrium 1 cm away from the cervix. The injection site was sutured, and the wound was closed with a 1 to 0 suture.

Six animals in the normal control group were randomly selected; they did not receive anesthesia or surgery. For the saline group, six rabbits received an injection of 0.5 mL of normal saline into the muscularis mucosae of the myometrium in place of the VX2 solution.

### Sample collection and pathological examination

At 15, 18, 21, 24, 27, and 30 days post-injection of the VX2 solution, rabbits were sacrificed by aeroembolism (injection of air into the ear vein) (*n* = 6 per time point). At 30 days, animals in the normal control and saline groups were sacrificed by the same method. EC and RLN tissues were observed macroscopically, and the long diameter (*a*) and short diameter (*b*) were measured to calculate EC and RLN volume using the following equation: *V* = *a* × *b*^2^/2. Under aseptic conditions, the uterus, EC, and RLNs were collected. The tissue was fixed in 10% formalin and embedded in paraffin. Sections were obtained and stained with hematoxylin and eosin (H&E). The stained EC and metastatic RLN tissues were observed under a light microscope by two independent pathologists.

### Statistical analyses

Data are presented as means ± standard deviation (SD). All statistical assessments were two-sided and evaluated at the 0.05 level of significant difference. Statistical analyses were performed using SPSS 15.0 statistics software (SPSS Inc, Chicago, IL, USA).

## Results

### Establishment of an animal model of EC with RLN metastasis

**Figure 1:**
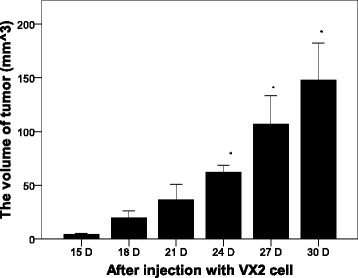
**Tumor volumes over time after injection with VX2 tumor cells.** The size of tumors in the experimental group was determined at the indicated time points.

**Figure 2:**
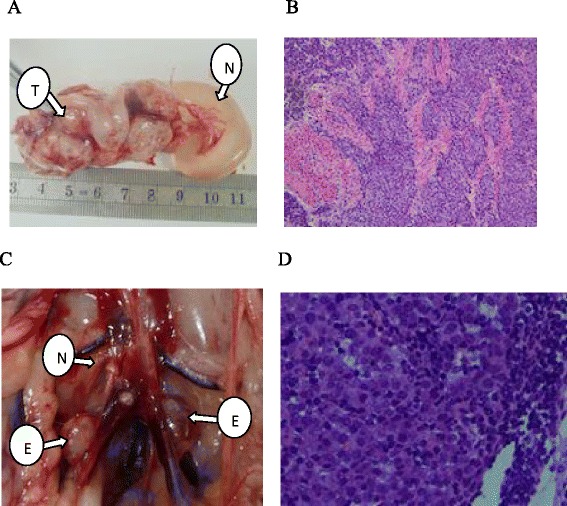
**Macroscopic and pathological analysis of rabbit endometrium and RLNs. (A)** EC at day 21. N, normal uterine body; T, endometrial tumor. **(B)** Histological examination of the endometrium at day 21 revealed orthotopic EC (H&E, 200×). **(C)** Retroperitoneal lymph node metastasis at day 21. E, enlarged RLN; N, normal RLN size. **(D)** Histological examination of RLN at day 21 revealed the presence of tumor cells (H&E, 400×).

All rabbits developed VX2 endometrial carcinoma which were confirmed with pathological examination .As shown in Figure 1, significantly increased tumor volume was observed at days 24, 27, and 30 post-injection of VX2 cells (*P* < 0.05). A representative image of the normal and tumor endometrium after 21 days is shown in Figure 2A. Histological analysis of the tumor tissue confirmed the presence of tumor cells (Figure 2B).

The presence of RLN metastasis was also assessed. RLN enlargement was not observed macroscopically at 15 days post-VX2 cell injection, and pathological examination confirmed the absence of RLN metastasis (data not shown). However, at days 18 and 21 post-VX2 cell injection, enlargement of several RLNs was observed (Figure 2C); however, pathological examination revealed the absence of RLN metastasis at the 18-day time point (data not shown). The presence of metastasis into several RLNs was observed at 21 days post-injection (Figure 2D). Enlargement of all RLNs and metastasis was noted at days 24, 27, and 30 post-injection.

## Discussion

The VX2 cell line is composed of squamous cell carcinoma cells derived from Shope virus-induced papilloma in rabbit [5]. Their high survival rates make them a suitable candidate for *in vivo* inoculation [5], which has been carried out in the liver, lung, uterus, and breast tissues to establish the corresponding animal models [6-8].

Domestic scholars confirmed by comparing different means of transplantation that the successful rate of tumor formation was high by tissue mass suspension injection [9]. Due to small tissue blocks, the tumor cells can obtain adequate nutrition and oxygen supply and the concentration of tumor cells resists the barrier of immunity, delaying the cleanup process, to ensure the normal growth of the tumor cells. With high rate of tumor formation and rapid growth, short modeling time can be reduced. This experiment confirmed that *model of EC with RLN metastasis* can be established within a month.

In the endometrium, inoculation of VX2 cells induced EC with lymph node metastasis after 14 to 21 days post-injection [6,7], which is similar to the results of the present study. These previous studies employed blocks of VX2 cells in the myometrium using microsurgical instruments while the present study introduced the VX2 cells via an injection, which removes the dependence on microsurgical instruments, that is less difficult and may increase the success rate of establishing the model as was evident in the 100% success rate of establishing EC and RLN metastasis observed in this study. The high success rate of this study using this method is consistent with results reported by Chen *et al*. [8]. Use of VX2 cells also induced EC more quickly than that reported for a mouse model of EC with lymph node metastasis that required 8 weeks to establish [10].

## Conclusions

Thus, an animal model of EC with RLN metastasis was established. Experiments with the model, not only from the biological level of endometrial cancer response but also to various treatments, such as chemotherapy drug screening and the selection of the optimal chemotherapy regimen, can also research the relationship between molecular markers from the molecular level with lymph node metastasis and micrometastasis. The current hot spot ‘molecular cancer staging,’ combining with the theory and technology of molecular biology and clinical staging of tumor to diagnose micrometastasis lesions in the body, which can make the stage of the tumor be determined more accurately, guides the determination of clinical treatment programs and reduces recurrence and distant metastasis. Therefore, this model provides a good repeatability large animal model for experimental study which is bound to have a major significance for the study of endometrial tumors.
